# Uses of Botulinum Toxin in Headache and Facial Pain Disorders: An Update

**DOI:** 10.3390/toxins17070314

**Published:** 2025-06-21

**Authors:** Pedro Augusto Sampaio Rocha-Filho, Moises Dominguez, Christopher L. Robinson, Sait Ashina

**Affiliations:** 1 Division of Neuropsychiatry, Centro de Ciências Médicas, Universidade Federal de Pernambuco (UFPE), Recife CEP 50670-901, PE, Brazil; 2 Headache Clinic, Hospital Universitário Oswaldo Cruz, Universidade de Pernambuco (UPE), Recife CEP 50100-130, PE, Brazil; 3 Department of Neurology, Weill Cornell Medicine, New York, NY 10021, USA; mod9040@med.cornell.edu; 4 Department of Anesthesiology, Perioperative and Pain Medicine, Brigham and Women’s Hospital, Boston, MA 02118, USA; christopherrobinsonmdphd@outlook.com; 5 Department of Neurology, BIDMC Comprehensive Headache Center, Harvard Medical School, Beth Israel Deaconess Medical Center, Boston, MA 02215, USA; sashina@bidmc.harvard.edu; 6 Department of Anaesthesia, Critical Care and Pain Medicine, BIDMC Comprehensive Headache Center, Harvard Medical School, Beth Israel Deaconess Medical Center, Boston, MA 02215, USA

**Keywords:** botulinum toxins, botulinum toxins, type A, therapeutics, headache, headache disorders, primary, headache disorders, secondary, migraine disorders, trigeminal neuralgia, post-traumatic headache, cluster headache, trigeminal autonomic cephalalgias

## Abstract

Botulinum toxin is a neurotoxin that is used in the treatments for several medical conditions, such as dystonia, spasticity, hemifacial spasm, overactive bladder, and hyperhidrosis. This toxin can potentially treat several pain disorders through botulinum toxin’s ability to inhibit the release of pro-nociceptive neurotransmitters into the synaptic cleft and its possible action on the central nervous system. This narrative review addresses the use of botulinum toxin in treating primary and secondary headaches and facial pain disorders. The highest level of evidence supporting its use varies among the headache and facial pain disorders: chronic migraine (multicenter, double-blind, placebo-controlled studies), trigeminal neuralgia (double-blind, placebo-controlled studies), post-traumatic headache (double-blind, placebo-controlled study), cluster headache (open-label clinical trials), nummular headache (open-label clinical trial), headache attributed to craniocervical dystonia (prospective cohort study), new daily persistent headache (retrospective cohort study), hemicrania continua, and SUNCT and SUNA (case reports). The site of toxin application and the doses used vary among the studies and depending on headache type. Botulinum toxin has been shown to be safe in different studies, with generally mild adverse reactions.

## 1. Introduction

Botulinum toxin is a neurotoxin produced by *Clostridium botulinum*. Although there are seven serotypes (A to G), only serotypes A (BoNT-A) and B are used in clinical practice [1,2]. Botulinum toxin inhibits the release of neurotransmitters and neuropeptides from the neuron into the synaptic cleft by targeting synaptosomal-associated protein-25 (SNAP-25) [3,4]. SNAP-25 is an essential component of the SNARE complex (soluble N-ethylmaleimide-sensitive fusion-attachment protein receptor), which facilitates the exocytosis of synaptic vesicle contents. At the motor neuron level, botulinum toxin inhibits acetylcholine release, impairing muscle contraction. This supports its use in the treatment of dystonia, spasticity, and cosmetic procedures [1,5]. Furthermore, botulinum toxin also prevents the release of nociception-associated neurotransmitters and neuropeptides such as calcitonin-gene-related peptide (CGRP), substance P, serotonin, glutamate, gamma-aminobutyric acid (GABA), noradrenaline, dopamine, enkephalin, and glycine from the neuron by cleaving soluble N-ethylmaleimide-sensitive factor attachment protein receptors. It also inhibits the surface expression of nociceptive receptors (TRPV1, TRPM8, TRPA1, P2X3, and GABA-A) and modulates inflammatory cytokines, contributing to its analgesic effects [1,5]. Studies with animal models show improvements in both nociceptive and neuropathic pain after the use of BoNT-A [4,6].

While botulinum toxin exerts its effects on peripheral nerves, it may also modulate pain processing in the central nervous system. This may be explained by a possible transsynaptic transport from nerve terminals to adjacent neurons and glial cells. There is also axonal transport mediated by axonal organelles traveling along microtubules [5].

The estimated global prevalence of an active headache disorder is 52.0% (95%CI 48.9–55.4) [7]. In addition to this high prevalence, headache disorders have a high impact on the lives of those who suffer from them, their families, and society [8,9,10,11,12,13,14,15,16,17,18].

Migraine is a particularly disabling neurological disorder, ranking as the second leading cause of years lived with disability, and the leading cause of disability in women between the ages of 15 and 49 per the World Health Organization [18]. Migraine is the third most disabling neurological disease in the United States, accounting for 705 years lived with disability per 100,000 Americans [19]. Although headache is the symptom that is generally assessed when assessing disability, other migraine symptoms such as cognitive impairment, photophobia, phonophobia, osmophobia, nausea, and vomiting also contribute to this disability. Some of these symptoms may occur during or between migraine attacks [20,21].

Trigeminal autonomic cephalalgias also have a significant impact. Patients with cluster headache have a worse quality of life in comparison with those without this disease and patients with migraine [9]. Of patients with cluster headache, 17% reported having lost their jobs because of the disease, and 25% reported a significant decrease in their ability to participate in social activities, family life, and household chores [22].

The decrease in productivity and the costs of medical care and treatment of headaches lead to a high economic impact. Given the disabling nature of headache disorders and their significant impact on patients, families, and society, effective treatments are needed to reduce this burden. Preventive headache treatments are used to reduce the frequency, severity, duration, and disability associated with particular headache disorders [8,9,10,11,12,13,14,18].

**Figure 1:**
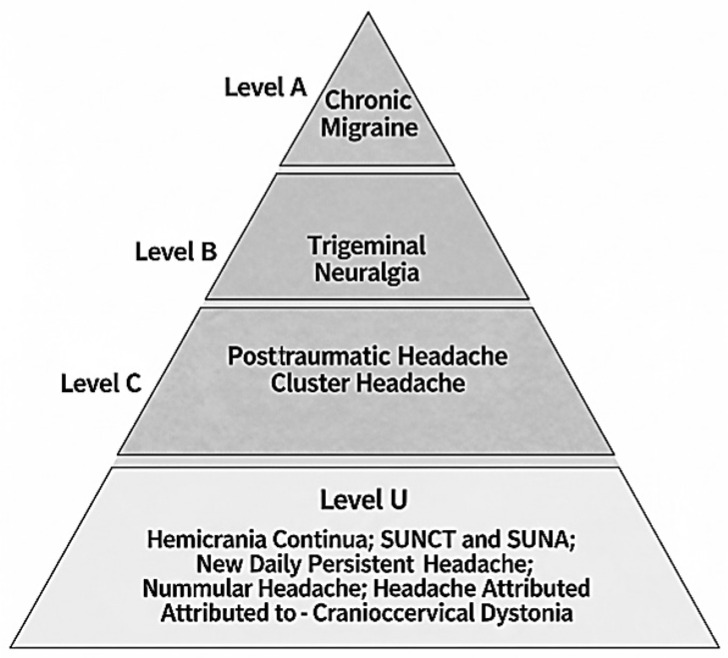
Degree of evidence for the use of botulinum toxin A for the treatment of headaches. Level A: established as effective; Level B: probably effective; Level C: possibly effective; Level U: the treatment is not proven.

This narrative review aims to discuss the use of botulinum toxin for the preventive treatment of primary and secondary headaches. Table 1 summarizes the main studies included. Figure 1 shows the degree of evidence for the use of botulinum toxin A for the treatment of headaches.

## 2. Method

This is a narrative review. A literature review was conducted on the PubMed platform in May 2025 using the following terms: “headache AND botulinum toxins AND therapeutics”; “migraine disorders AND botulinum toxins AND therapeutics“; “trigeminal autonomic cephalalgias AND botulinum toxins AND therapeutics”; “cluster headache AND botulinum toxins AND therapeutics”; “hemicrania Continua AND botulinum toxins AND therapeutics”; “SUNCT Syndrome AND botulinum toxins AND therapeutics”; “New Daily Persistent Headache AND botulinum toxins AND therapeutics;” “nummular headache AND botulinum toxins AND therapeutics”; “headache disorders, secondary AND botulinum toxins AND therapeutics”; “Dystonia AND headache AND botulinum toxins AND therapeutics”; “post-traumatic headache AND botulinum toxins AND therapeutics”; “trigeminal neuralgia AND botulinum toxins AND therapeutics”. All articles considered to be relevant were included.

Evidence from the literature was assessed according to the levels of evidence and recommendation classification defined by the American Academy of Neurology`s guidelines [39]. Levels of evidence for the use of botulinum toxin were considered: Level A (established as effective, ineffective or harmful) when there are at least 2 class I studies; Level B (probably effective, ineffective or harmful) when there is at least 1 class I study or 2 class II studies; Level C (possibly effective, ineffective or harmful) when there is at least 1 class II study or 2 class III studies; Level U (the treatment is not proven) when there is inappropriate or conflicting data.

## 3. Chronic Migraine

Chronic migraine, considered more disabling than episodic migraine, is characterized by having 15 or more headache days per month, with at least 8 days of those days being migraine days, persisting for more than three months [40].

The PREEMPT (Phase III REsearch Evaluating Migraine Prophylaxis Therapy) studies demonstrated that BoNT-A injections are effective in the treatment of chronic migraine. In the PREEMPT protocol, 155 U of onabotulinum toxin A are applied to 31 fixed sites across seven head/neck muscle areas. At the investigator’s discretion, an additional 40 U could be administered into the temporalis, occipitalis, and/or trapezius muscles using a follow-the-pain strategy. The maximum total dose was 195 U at 39 sites. Both studies had a baseline period of 28 days to collect headache data on a headache diary and a post-intervention follow-up of 24 weeks with two cycles of application of onabotulinum toxin A or placebo of 12 weeks each [23,24].

The PREEMPT 1 study was conducted across 56 centers in North America and included 679 patients who were randomized to receive onabotulinum toxin A or placebo. The primary endpoint was the mean change from baseline in headache episode frequency at week 24 and secondary endpoints included was the mean change in headache and migraine days from baseline to the 28 days ending with week 24. Although there was no statistically significant difference between the groups regarding the primary outcome, the onabotulinum toxin A group showed a significantly greater decrease in the mean number of headache days compared to the placebo group [23]. The PREEMPT 2 study involved 66 centers in North America and Europe and included 705 patients. There was a significantly greater decrease in headache days in the group that used BoNT-A than in the placebo group (primary outcome). BoNT-A was also significantly better than placebo in secondary outcomes such as decreasing migraine days, moderate–severe headache days, triptan intake frequency, and decreasing headache impact [24]. The open-label follow-up of these studies showed that onabotulinum toxin A is safe and had a sustained effect over 56 weeks of follow-up [41].

A systematic review evaluated the use of BoNT-A for the treatment of episodic migraine and chronic migraine. Only one clinical trial for episodic migraine was included, which found no difference in the number of days with migraine between those who received BoNT-A or placebo. A meta-analysis of four studies for chronic migraine found a decrease of 3.1 migraine days per month (95% CI: −4.7 to −1.4) in favor of those who used BoNT-A [42].

Real-world studies also demonstrate the benefits of using onabotulinum toxin A in the treatment of chronic migraine. A systematic review of 44 studies published between 2010 and 2021 showed a decrease of 10.6 headache days per month (95% CI: –12.31, –8.97) after 24 weeks of follow-up and 10 fewer days (95% CI: –14.92, –5.73) after 52 weeks. The prospective studies showed that 39% (95% CI: 22%, 55%) of patients had a decrease in headache frequency greater than or equal to 50% [43].

A study evaluated pooled data from 16 European headache centers that had conducted real-world studies on the use of onabotulinum toxin A for the treatment of chronic migraine. A total of 2879 patients were included and followed for at least 9 months [44,45,46]. Both men and women had a significant decrease in headache frequency and use of pain medications after treatment [45]. Comparing those who had excellent responses (reduction in the frequency of headache days ≥75% or headache frequency less than 4 days per month) with non-responders, the former had a higher prevalence of medication overuse at baseline and a higher proportion of excellent responses in the first and second cycles of BoNT-A [44]. Among the patients included, 235 were elderly. There was no difference in response to treatment, the percentage of patients who discontinued treatment, or the incidence of adverse events when comparing elderly and non-elderly patients. No serious adverse events were reported [46].

A recent systematic review and meta-analysis evaluated the use of onabotulinum toxin A for the prevention of chronic migraine in children and adolescents and included 14 studies (2 clinical trials and 12 observational studies) [47]. The meta-analysis of the observational studies showed a decrease in headache intensity and frequency in chronic migraine after treatment. Only one class 1 study was included in this systematic review [47]. This class 1 study evaluated patients aged 12 to 17 years with chronic migraine and compared onabotulinum toxin A 155 U (n = 45), 74 U (n = 43), or placebo (n = 37). The study had a 4-week baseline to collect headache data on a headache diary and a 12-week follow-up. All three groups improved in the frequency of headache days after the intervention, with no difference between the groups. Most adverse effects were mild and none of the participants discontinued treatment due to adverse effects [48]. Thus, onabotulinum toxin A for the treatment of chronic migraine in children and adolescents faces the same difficulty encountered with other types of treatments, which is the large placebo effect in this age group.

Clinical trials evaluating the use of BoNT-A for chronic migraine have excluded pregnant people from the studies. A recent study used the Allergan Global Safety Database and included 397 pregnancies in which the pregnant people had used BoNT-A (95% in the first trimester or before the estimated date of conception) for various reasons (30% for migraine/headache). Among the pregnant people followed prospectively, the incidence of fetal loss was 19%, and of major fetal defects was 0.7%. These values were considered within the expected range for the general population of the same age [49]. Another study prospectively followed 126 pregnant people who were using onabotulinum toxin A for chronic migraine, 97 of whom continued treatment during pregnancy. There were no fetal malformations. There were three (2.4%) fetal losses, a value that was considered lower than expected for the general population [50]. The International Headache Society has recently recommended that onabotulinum toxin A may be an option for pregnant people due to its limited systemic effects; however, the risks and benefits must be carefully weighed [51].

## 4. Cluster Headache

Cluster headache is the most common trigeminal autonomic cephalalgia characterized by attacks of severe, strictly unilateral pain, lasting from 15 to 180 min [40]. The pain is associated with ipsilateral conjunctival injection, lacrimation, nasal congestion, rhinorrhoea, forehead and facial sweating, miosis, ptosis, and/or eyelid edema, and/or restlessness or agitation. In chronic cluster headache, pain attacks occur without a remission period or with remissions lasting less than 3 months in a year [40].

The evidence supporting the use of BoNT-A for treating cluster headache primarily comes from small, open-label clinical trials [25,26,52,53]. Most of these studies were conducted with patients with refractory chronic cluster headache. The sites and methods of application varied between studies.

The first of these studies included twelve patients, of whom nine had chronic cluster headache. A total dose of 50 U of onabotulinum toxin A was applied ipsilateral to the headache site (temporalis muscle: 10 U, frontalis: 10 U, splenium capitis: 10 U and trapezius: 20 U). The patients were followed for 90 days. There was an improvement in headache frequency and intensity compared to baseline in three patients who had chronic cluster headache [52].

Lampl et al. [25] evaluated the PREEMPT treatment paradigm for the treatment of refractory chronic cluster headache. Twelve patients were included and followed for 24 weeks after the application of onabotulinum toxin A. There was a significant reduction in the frequency of headache days (- 16 days; 95% CI: −21; −12), total headache duration (- 1.3 min; 95% CI: −1.8; −878), and headache intensity compared to baseline. Of the patients, 59% experienced a reduction of more than 50% in the total duration of headache compared to baseline [25].

Blatbak et al. [26] evaluated sphenopalatine ganglion blockade with 25 to 50 U of onabotulinum toxin A ipsilateral to the headache for refractory chronic cluster headache. A surgical navigation device was used to perform the injection. Ten patients were followed for 24 weeks, with seven patients were included in the analysis. There was a decrease in the frequency of headache attacks compared to baseline at months 1, 2, 3, 4, and 6 after the procedure. A ≥ 50% reduction in cluster headache attack frequency was observed in five of the seven patients [26]. During the follow-up with these patients, seven had repeat injections as needed. There was a decrease in the frequency of attacks (month 18: *p* = 0.018; month 24: *p* = 0.018), the frequency of severe and unbearably intense attacks (month 18: *p* = 0.018; month 24: *p* = 0.028), and a significant increase in days without headache attacks at months (*p* = 0.027; month 24: *p* = 0.018) 18 and 24 after treatment compared to baseline [53]. A series of 31 patients with refractory chronic cluster headache who underwent the same procedure was subsequently published. There was a decrease in the frequency of attacks compared to baseline after each one of the four applications performed (*p* = 0.002; *p* = 0.006; *p* = 0.028; *p* = 0.018). The procedure proved to be safe, with 93% of the adverse reactions presented classified as mild such as pain or swelling and chin numbness [54].

The same group conducted an open clinical trial to evaluate the effectiveness of blockade of the ipsilateral otic ganglion with onabotulinum toxin A (12.5 and 25 international units) for intractable chronic cluster headache [55]. Surgical navigation was also used to perform the injections. Ten patients were enrolled and followed for 6 months. While the treatment showed a favorable safety profile, it did not prove to be effective [55].

## 5. Hemicrania Continua

Hemicrania continua is an indomethacin-responsive trigeminal autonomic cephalalgia characterized by a strictly unilateral headache and continuous or long-lasting attacks, occurring for more than 3 months [40]. The headache is associated with autonomic symptoms ipsilateral to the pain that occurs during periods of headache exacerbation [40].

Eleven cases of hemicrania continua have been reported to demonstrate an improvement in headache intensity and frequency after treatment with BoNT-A [27,56,57]. In ten of these cases, BoNT-A application followed the PREEMPT protocol. The reason for using BoNT-A was either the inability to tolerate indomethacin due to adverse effects, or headache recurrence after a period of control with indomethacin [27,56,57].

## 6. SUNCT and SUNA

Short-lasting unilateral neuralgiform headache attacks with conjunctival injection and tearing (SUNCT) and short-lasting unilateral neuralgiform headache attacks with cranial autonomic symptoms (SUNA) are characterized by moderate–severe, side-locked headache attacks, typically affecting the first and second division of the trigeminal nerve [40]. These headache attacks occur as a series of stabs or in a saw-tooth pattern, lasting 1–600 s, and associated with ipsilateral cranial autonomic symptoms [40].

There are three case reports demonstrating a favorable outcome with the use of BoNT-A in SUNCT and SUNA [28,29,30]. In one of report, a male adolescent with SUNCT received BoNT-A subcutaneously into the area of pain (2.5–5 U at each site, separated by 1.5 cm) [28]. This patient had no recurrence of pain at 17 months [28]. In the other case, a 55-year-old man had treatment refractory SUNCT with a partial response to lamotrigine and gabapentin. Onabotulinum toxin A was injected at four points around the orbit, injecting 10 U at each site, and repeated every 3 months. There was a significant improvement in the frequency and intensity of attacks within a few weeks. The patient remained stable over a follow-up period of two and a half years, with lamotrigine and gabapentin continued throughout [30].

A case report demonstrated an improvement in SUNA symptoms with BoNT-A in a 65-year-old woman. BoNT-A was injected subcutaneously in the area of pain (2.5–5 U at each site, separated by 1.5 cm. Total: 100 U). Following treatment, there was an improvement in the intensity and frequency of attacks. A second treatment cycle of 50 U of BoNT-A was administered one month later. The patient remained pain-free during the 16 months of follow-up [29].

## 7. New Daily Persistent Headache

New daily persistent headache is a primary headache disorder characterized by the clear memory of the onset of pain, which becomes continuous and unremitting within a 24 h period [40]. The headache must persist for three months or more [40]. Patients can often identify a precipitating event, the most common being viral respiratory conditions, stressful events, and extracranial surgeries requiring intubation [58,59,60]. This headache can have a chronic migraine or chronic tension type phenotype and is often refractory to treatment. There are currently no clinical trials evaluating its treatment [58,59].

Two cases of BoNT-A used for the treatment of new daily persistent headache have been described. In the first case, a total of 100 U of BoNT-A was applied to the glabellar, frontalis, temporalis, medial, and lateral suboccipital, semispinalis, splenius capitus, and trapezius muscles. The patient was followed for one year and remained headache-free for 8 to 10 weeks after each application cycle [61]. In the second case, a total of 195 units of BoNT-A were applied. There was a partial improvement, but the patient was never pain-free [62].

A retrospective cohort study of 19 patients with new daily persistent headache demonstrated headache improvement after treatment with onabotulinum toxin A. Data collection was performed by reviewing medical records and the onabotulinum toxin A application followed the PREEMPT protocol. There was an improvement in the frequency of headache attacks in 50% of patients after 6 months and in 64% after 12 months of treatment. There was an improvement in headache intensity in 15%, 50%, and 78% of patients after 3, 6, and 12 months of treatment, respectively [31].

## 8. Nummular Headache

Nummular headache is characterized by pain in a well-circumscribed area of the scalp, with a fixed size, shape, and contour, typically representing a coin [40]. The diameter of the painful area varies from one to six centimeters. The pain may be intermittent or continuous and have variable frequency, duration, and patterns. Usually, there is only one painful area, with the parietal region being the most frequent site. Rarely, two areas of pain may occur [63,64,65].

An open-label clinical trial evaluated onabotulinum toxin A for the treatment of patients with nummular headache. Fifty-three patients with a frequency of at least 10 headache days per month were included. Five units of onabotulinum toxin A were applied to five points in the painful area, creating a cross that included the largest diameter (total of 25 units). This application was repeated at 3 months and the follow-up was 24 weeks. There was a significant improvement in headache frequency (primary outcome; baseline: 24.5 days; week 20–24: 6.9 days; *p* < 0.001), days with severe pain, and days with the use of acute pain medications when comparing weeks 20 to 24 with baseline [32].

## 9. Post-Traumatic Headache

To classify a post-traumatic headache as “headache attributed to traumatic injury to the head,” it must begin within seven days of the trauma, recovery of consciousness after the trauma, or discontinuation of medications impairing the ability to sense or report headache following the injury to the head [40]. If the headache resolves within 3 months or the headache has not yet resolved but three months have not yet passed since its onset, it is classified as acute headache attributed to traumatic injury to the head. If the headache persists for more than three months, it is classified as a persistent headache attributed to traumatic injury to the head [40].

A double-blind, randomized, placebo-controlled, cross-over clinical trial evaluated Abobotulinum toxin A for the treatment of persistent headache attributed to traumatic injury to the head [33]. Forty patients were included and the PREEMPT protocol was used. Patients were followed for 32 weeks. The primary outcome was the change in headache days in relation to baseline. There was a significantly greater decrease in pain frequency (−1.6 headache days/week; 95% CI: −0.6; −2.6 vs. + 0.01 headache days/week; 95% CI: −0.06; + 0.08; *p* = 0.48) and intensity (−0.06 points/week; 95% CI: −0.1; −0.11 vs. + 0.04 points/week; 95% CI: −0.01; + 0.08; *p* = 0.006) in the BoNT-A group than in the placebo group [33].

## 10. Headache Attributed to Craniocervical Dystonia

Dystonia is a movement disorder characterized by sustained or intermittent muscle contractions that lead to abnormal, often repetitive movements and postures. These movements are typically patterned and twisting [66,67]. Craniocervical dystonias include pharyngeal dystonia, spasmodic torticollis, mandibular dystonia, lingual dystonia, and a combination of cranial and cervical dystonias [40,68]. Sixty percent of patients with craniocervical dystonia have headache. [68]

In addition to the presence of craniocervical dystonia, to classify a headache as headache attributed to craniocervical dystonia (HACCD), there must be at least two of the following evidence of causation: (1) pain has developed in temporal relation to the onset of craniocervical dystonia; (2) pain has significantly worsened in parallel with the progression of the craniocervical dystonia; (3) pain has significantly improved or resolved in parallel with improvement in or resolution of the craniocervical dystonia; (4) pain location corresponds to the location of the dystonic muscles [40,68].

Although there are no clinical trials specifically evaluating the treatment of HACCD, some studies have shown that BoNT-A treatment for dystonia can improve associated headaches [69,70,71,72,73]. However, these studies did not directly assess BoNT-A’s efficacy in HACCD [69,70,71,72,73]. A prospective cohort study demonstrated that treatment of dystonia with BoNT-A decreased the impact of the HACCD but did not affect other headache types experienced by patients with cervical dystonia. In this study, the impact of headaches was measured by the Headache Impact Test (HIT-6). It is unclear whether headache improvement in this condition occurs by the effect of BoNT-A on pain or by improving dystonia [34].

## 11. Trigeminal Neuralgia

Trigeminal neuralgia is characterized by unilateral, electric-shock-like, shooting, stabbing, or sharp pain paroxysms in one or more divisions of the trigeminal nerve [17]. These last from fractions of a second to up to two minutes and can be triggered by innocuous stimuli in the affected trigeminal distribution [40].

Open-label clinical trials have shown that the use of BoNT-A for the treatment of trigeminal neuralgia is effective and safe [74,75,76,77,78,79,80,81]. Four studies that included 8 to 15 patients showed an improvement in the intensity [74,75,76,77] and frequency of pain [74,75,76,77]. These results were corroborated by other larger studies that included 40 to 100 patients and showed an improvement in the intensity [78,79,80,81] and frequency of pain [78]. This improvement occurred in both patients with paroxysmal pain and those with continuous pain [78]. The method of application as well as the dose of BoNT-A varied between the studies, with the application generally being made transcutaneously (subdermally), at the site of pain and in the trigger zones [74,75,76,77,78,79,80,81]. In one of the studies, the injection was made in the sphenopalatine ganglion on the same side as the pain [75].

Four double-blind, randomized, placebo-controlled studies evaluated the use of BoNT-A for the treatment of trigeminal neuralgia and included twenty [35], thirty-six [36], forty-two [37], and eighty-four patients [38]. The injections were made subcutaneously or intradermally and/or submucosally in the area of pain, and the doses were 50 U (plus 10 U in the masseter muscle if the third branch of the trigeminal nerve was involved) [36], 40 to 60 U [35], 25 and 75 U [38], and 75 U [37], respectively. The follow-up period was 8 to 12 weeks [35,36,38]. After the intervention, the intensity [35,36,37,38] and frequency [35,37] of pain were significantly lower in the BoNT-A group than in the placebo group, and only mild–moderate adverse reactions were recorded [35,36,37,38].

These studies were classified according to quality using the Jadad score (zero = poor; five = good): Shehata et al. (score = 5); Wu et al. (score = 4); Zhang et al. (score = 2); and Zuniga et al. (score = 3) [82]. The four studies defined their primary outcomes a priori, but did not calculate sample size and therefore may not have had the power to show small differences. Despite being small studies and having different methods (selection criteria, doses, forms of BoNT-A application, follow-up period), all showed benefits with the use of BoNT-A.

## 12. Ongoing Clinical Trials

Ongoing clinical trials were identified through the website https://clinicaltrials.gov. In this process, this website was accessed in June 2025 and entered “Condition/disease”: “headache” and “Intervention/treatment”: “Botulinum toxin”. All active clinical trials were considered. Table 2 shows these studies. Clinical trials on migraine, chronic cluster headache and post-traumatic headache were found. These clinical trials are expected to end in 2025 and 2026.

## 13. Conclusions

BoNT-A has been shown to be used to treat a variety of headache disorders, with more evidence available for chronic migraine. The level of evidence supporting this use varies by headache disorder: chronic migraine and trigeminal neuralgia (double-blind, placebo-controlled studies), post-traumatic headache (double-blind, placebo-controlled study), cluster headache (open clinical trials), nummular headache (open clinical trial), headache attributed to craniocervical dystonia (prospective cohort study), new daily persistent headache (retrospective cohort study), hemicrania continua, and SUNCT and SUNA (case reports). The site, methods of application, and doses used vary between studies and according to the type of headache, which makes it difficult to compare different studies. BoNT-A was reported to be safe in different studies, with adverse effects generally being mild.

## Figures and Tables

**Table 1:** Description of the main studies included that evaluate the treatment of headaches with botulinum toxin A.

Author/Year	Number of Participants	Study Design	Type of Botulinum Toxin	Site of Injections	Follow Up After the Intervention	Main Findings
	**Chronic Migraine**					
Aurora et al., 2010 [23]	679	Multicenter, double-blind, placebo-controlled clinical trial	Onabotulinum toxin A	PREEMPT protocol	24 weeks	Greater decrease in the mean number of headache days than the placebo group.
Diener et al., 2010 [24]	705	Multicenter, double-blind, placebo-controlled clinical trial	Onabotulinum toxin A	PREEMPT protocol	24 weeks	Greater decrease in the mean number of headache days than the placebo group.
	**Chronic Cluster headache**					
Lampl et al., 2018 [25]	12	Open-label clinical trial	Onabotulinum toxin A	PREEMPT protocol	24 weeks	Reduction in the frequency of headache days, total headache duration, and headache intensity compared to baseline.
Blatbak et al., 2016 [26]	10	Open-label clinical trial	Onabotulinum toxin A	Sphenopalatine ganglion blockade	24 weeks	Decrease in the frequency of headache attacks compared to baseline.
	**Hemicrania continua**					
Miller et al., 2015 [27]	9	Case series	Onabotulinum toxin A	PREEMPT protocol	-	Improvement in headache intensity and frequency.
	**SUNCT and SUNA**					
Zhang et al., 2016 [28] Zhang et al., 2019 [29] Zabalza, 2012 [30]	3	Case report	Botulinum toxin A	Subcutaneously into the area of pain	16 to 30 months	Improvement in headache intensity and frequency.
	**New daily persistent headache**					
Ali et al., 2019 [31]	19	Retrospective cohort study	Abobotulinum toxin A	PREEMPT protocol	12 months	Improvement in headache intensity and frequency.
	**Nummular headache**					
García-Azorín et al., 2019 [32]	53	Open-label clinical trial	Onabotulinum toxin A	Area of pain	24 weeks	Improvement in headache intensity and frequency.
	**Post-traumatic headache**					
Zirovich et al., 2021 [33]	40	Double-blind, placebo-controlled clinical trial	Abobotulinum toxin A	PREEMPT protocol	32 weeks	Greater decrease in headache intensity and frequency than the placebo group.
	**Headache attributed to craniocervical dystonia**					
Eugenio Ramalho Bezerra and Sampaio Rocha-Filho, 2020 [34]	24	Prospective cohort study	Abobotulinum toxin A	Dystonic muscles	16 weeks	Decrease in headache impact.
	**Trigeminal neuralgia**					
Shehata et al., 2013 [35]	20	Double-blind, placebo-controlled clinical trial	Onabotulinum toxin A	Area of pain	12 weeks	Greater decrease in headache intensity and frequency than the placebo group.
Zúñiga et al., 2013 [36]	36	Double-blind, placebo-controlled clinical trial	Onabotulinum toxin A	Area of pain	12 weeks	Greater decrease in headache intensity than the placebo group.
Wu et al., 2012 [37]	42	Double-blind, placebo-controlled clinical trial	Botulinum toxin A (made in Lanzhou Biological Products Institute)	Area of pain	12 weeks	Greater decrease in headache intensity and frequency than the placebo group.
Zhang et al., 2014 [38]	84	Double-blind, placebo-controlled clinical trial	Botulinum toxin A (made in Lanzhou Biological Products Institute)	Area of pain	8 weeks	Greater decrease in headache intensity than the placebo group.

**Table 2:** Ongoing clinical trials evaluating botulinum toxin for the treatment of headaches.

Author/Sponsor	ClinicalTrials.gov ID	Headache	Study Design	Intervention	Site of Injections	Estimated Study Completion Date
Zagazig University, Egypt	NCT06684249	Chronic migraine	Randomized controlled trial	Supratrochlear and greater occipital nerve block (local anesthetics and corticosteroids) vs. onabotulinum toxin A	PREEMPT protocol	10 July 2025
Ipsen Clinical Study Enquiries	NCT06047444	Chronic migraine	Multicenter, double-blind, placebo-controlled clinical trial	Dysport	“muscles across the head, neck, face and shoulders.”	21 December 2026
Cairo University, Egypt	NCT06974617	Chronic migraine	Randomized, controlled trial	Onabotulinum toxin A vs. sphenopalatine ganglion block (lidocaine)		1 October 2025
Navy Medical Center at Camp Lejeune, United States	NCT05598723	Chronic migraine	Randomized, double-blind, controlled trial	Onabotulinum toxin A (Botox) vs. incobotulinum toxin A (Xeomin)		24 August 2026
Fondazione Policlinico Universitario Campus Bio-Medico, Italy	NCT06537700	Chronic migraine	Open-label clinical trial	Onabotulinum toxin A		31 December 2025
Ki Health Partners. LLC. United States	NCT06154070	Migraine	Prospective, multicenter, unblinded study	Doxibutlinum toxin A	“EEG paradigm”	October 2024 (Active, not recruiting)
Ipsen Clinical Study Enquiries	NCT06047457	Episodic migraine	Multicenter, double-blind, placebo-controlled clinical trial	Dysport	“muscles across the head, neck, face and shoulders.”	21 December 2026
Norwegian University of Science and Technology, Norway	NCT03944876	Chronic cluster headache	Multicenter, double-blind, placebo-controlled clinical trial	Botulinum toxin type A	Sphenopalatine ganglion blockade	September 2025
Danish Headache Center	NCT06839118	Post-traumatic headache	Double-blind, placebo-controlled clinical trial	Onabotulinum toxin A	PREEMPT protocol	1 August 2026

## Data Availability

No new data were created or analyzed in this study.
